# Stress shielding effect after total hip arthroplasty varies between combinations of stem design and stiffness—a comparing biomechanical finite element analysis

**DOI:** 10.1007/s00264-023-05825-7

**Published:** 2023-06-03

**Authors:** Rene Burchard, Jan A. Graw, Christian Soost, Jan Schmitt

**Affiliations:** 1 University of Marburg, Marburg, Germany; 2 Department of Orthopedics and Trauma Surgery, University of Giessen and Marburg, Marburg, Germany; 3 Department of Orthopedics and Trauma Surgery, Lahn-Dill-Kliniken, Rotebergstr. 2, 35683 Dillenburg, Germany; 4 Department of Anesthesiology and Intensive Care Medicine, Ulm University Hospital, Ulm, Germany; 5 FOM University of Applied Sciences, Essen, Germany

**Keywords:** Stress shielding, Short stem, THA, Hip, Bone remodelling, Hollow stem

## Abstract

**Purpose:**

Total hip arthroplasty (THA) has become a highly frequent orthopaedic procedure. Multiple approaches have been made to design the femoral component for THA with a mechanical behaviour as close as possible to a natural femur. The aim of this study was to compare different combinations of design and biomechanical properties of THA prostheses and their impact on stress shielding of the periprosthetic bone.

**Methods:**

Virtual implantation of different stem designs (straight standard stem, straight short stem, anatomical short stem) by finite element analysis based on in vivo data from computer tomography was performed. For each stem, three grades of stiffness were generated, followed by a strain analysis.

**Results:**

Reduction of stem stiffness led to less stress shielding. Implantation of an anatomical short-stem prosthesis with low stiffness provided the most physiological strain-loading effect (*p* < 0.001).

**Conclusion:**

A combination of a short and an anatomically designed stem with a low stiffness might provide a more physiological strain transfer during THA. Biomechanical properties of the femoral component for THA should be considered as a multifactorial function of dimensions, design, and stiffness.

## Introduction

Total hip arthroplasty (THA) is one of the most significant milestones in orthopaedic surgery. First efforts to replace the femoral head with ivory were made in the nineteenth century. Since these early days, significant technological progress was made in implant development including advances like the Charnley®-prosthesis or the Zweymüller®-stem [1].

Because of the current demographic trend and increasing numbers of THA implantations, surgeons are challenged by an increasing number of complex revision procedures [2]. Therefore, the primary goal for further developments in THA is the longest possible survival of the bone-implant interfaces including the implants themselves. Apart from polyethylene abrasion, periprosthetic stress shielding with concomitant reduction of the bone mass is a well-known phenomenon which leads to non-physiological bone remodelling processes [2, 3].

The desire to preserve as much bone material as possible in THA, which provides an appropriate backup in case revision surgery is necessary, led to new implant designs and properties. Short-stem prostheses initiated by Morrey in 1989 have been designed for this reason [4]. So far, many studies have shown that these short-stemmed prostheses result in a significantly higher preservation of bone stock compared to standard prostheses [5–8].

An additional approach to mimic biomechanical properties of the hip with a THA was initiated by Morscher and Mathys with the so-called “isoelastic” composite hip stem [9]. However, problems with stem ingrowth and early failure of this prosthesis led to the disappearance of this implant from the market [10]. Based on the findings with the “isoelastic” composite hip stem, Gross and Abel investigated whether a hollow hip stem can provide a strain load situation close to a real bone [11]. They developed an optimised hollow stem structure in a theoretical approach. Other authors used similar approaches to design a femoral component of the THA that shows a physiological behaviour close to that of natural bone [12–15]. However, because of unwarranted or harmful side effects, none of these implants are currently used in clinical routine.

Each of the above studies examined only one characteristic effect after implantation of the respective hip stem prosthesis, either the influence of stem length or the influence of stem stiffness. No study looked at the combination of these two different parameters. To fill this knowledge gap, the combination of different implant properties was the subject of the present study. Based on a validated in vivo data set, virtual hip stem implantation within the framework of a finite element analysis (FEA) was performed [16]. Three typical types of stem designs combined with different stem stiffnesses were tested for their strain load and their consecutive effects on the femoral bone.

## Materials and Methods

### Ethics

The Medical Ethics Committee of the University of Marburg approved this study (number of ethical approval: 84/96). Written informed consent was obtained from all study participants before participation.

### Materials

The study is based on computed tomography (CT) data from a 75-year-old woman suffering from hip osteoarthritis. Validated data from her right femur were used for the analysis [16]. Characteristics of the three different stem prostheses including their specifications such as manufacturer, implanted size, size-specific length, largest depth of the stem body, and stem type are shown in Table 1.

**Table 1:** Specifications of all investigated stems

Stem	Manufacturer	Stem size	Stem type	Length	Max. thickness
Fitmore®	Zimmer®, Warsaw, USA	A4	Short, anatomical	93 mm	14 mm
Ecofit Short®	Implantcast®, Buxtehude, Germany	6.25	Short, straight	97 mm	12 mm
CLS Spotorno®	Zimmer®, Warsaw, USA	8	Standard, straight	146 mm	17 mm

### Methods

Analytical methods were chosen as described previously [17]. Scanner settings (Somatom® Plus-4, Siemens, Erlangen, Germany) and transformation of CT voxels to finite elements (FE) analysis were adjusted as described previously [16, 17].

To evaluate the impact of the different stem design/stiffness combinations on periprosthetic bone structure, a classic strain analysis for each combination of stem type and stem stiffness was conducted. Virtual implantation of the different stem designs was performed with FE software Ansys® (Ansys 14.5.7, Ansys Inc., Canonsburg, USA), and a geometrical matrix for each stem was generated according to Schmitt et al., 1997 [18]. It was assumed that stems showed a stable and rigid bonding with full calcar contact.

**Fig. 1:**
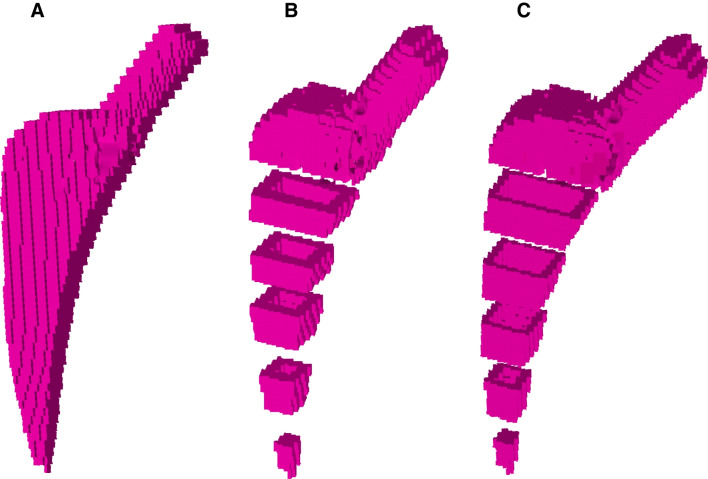
Virtually generated finite element models of the Fitmore®-prosthesis. The figure shows the modelling of the hollow stems with thinning shells. **A** Full-bodied original stem, **B** double-layer stem with a 1.32-mm big shell, and double-layer stem with a 0.66-mm small shell (**C**). Graphics were taken from Ansys®

Inspired by the full metal jacket principle of bullets, a double-layer hip stem with a titanium shell and a bone-marrow-like core was developed. A similar approach was realised in the form of the Epoch® prosthesis (Zimmer Inc, Warsaw, USA), however in different material layer order [19]. In our study, every stem was designed in three different phenotypes: full-body titanium (FB), double layer with a 1.32-mm big shell (BS), and double layer with a 0.66-mm small shell (SS). In FEA, the double layer concept was realised by assigning bone-marrow-like elastic modulus to the stem’s core (Fig. 1).

Based on the work of Pauwels and according to Stolk and colleagues, 347% of the body weight were applied as the resulting head force during strain loading [20, 21]. Three-dimensional directions of this force were defined according to Lengsfeld and colleagues [22]. Additional muscle forces were disregarded while those show a wide variance in vivo and produce no bias in case the same loads were used for each model [23].

During the solution process, the gradient solver (default settings) of the FE software was used for the strain simulation process after the hip centre force was applied [17]. Slice-by-slice analysis was followed by linear analysis with full resolution as described previously [11, 12, 17]. Periprosthetic regions of interest (ROI) were defined for each stem type accordingly [24].

### Statistical Analysis

Statistical analysis was performed with statistical software package SPSS® Version 24 (IBM, Armonk, North Castle, New York, USA). Groups were compared using the Kruskal–Wallis-H test with Dunn’s post hoc test and a Bonferroni correction for multiple testing [25]. The dependent variable strain energy density was calculated as integral of the area below the fitting curves divided into 20 equidistant sections.

## Results

### Full resolution analysis

Full-resolution analysis demonstrated advantages of the anatomical short stem with regard to the stress-shielding effect at the proximal femur.

**Fig. 2:**
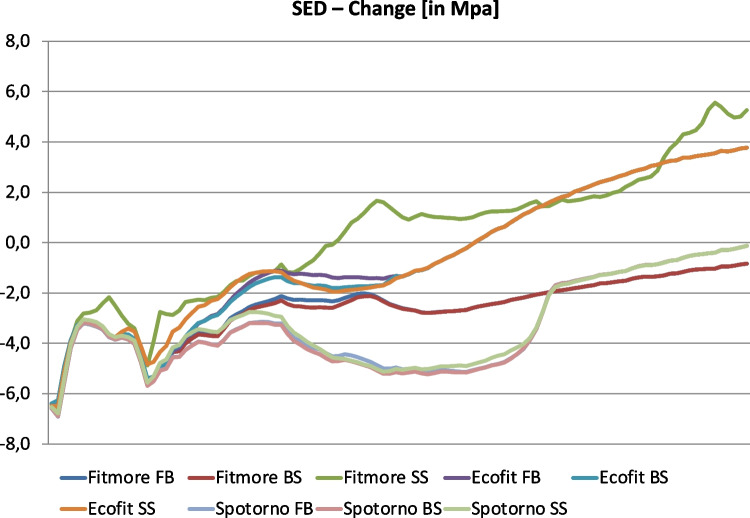
SED-Changes after virtual stem implantation [MPa]. *X*-axis is defined by the anatomical location starting from trochanteric tip and ends three centimetres below the CLS Spotorno® prosthesis tip. The different stem types (Fitmore® FB (blue), Fitmore® BS (red), Fitmore® SS (green), Ecofit Short® FB (violet), Ecofit Short® BS (petrol), Ecofit Short® SS (orange), CLS Spotorno® FB (light blue), CLS Spotorno® BS (light red), and CLS Spotorno® SS (light green)) were taken for stress analysis. Only the anatomical stem (Fitmore®) offers a reduced stress shielding effect by reducing its stiffness to a small shell (*p* < 0.001)

Figure 2 shows strain patterns of all 9 combinations in full resolution for the bone from the trochanteric tip to the diaphyseal area. Particularly in the metadiaphyseal area, short stems provide a biomechanical behaviour that is closer to human physiology compared to the behaviour of the standard stem. Strain load differed significantly between the three types of the Fitmore® stems (FB, BS, SS; *χ*^2^ = 20.04, *p* < 0.001). Type Fitmore® SS showed a reduced stress shielding effect compared to the Fitmore® FB (*p* < 0.001) while the stress shielding effect of the Fitmore® FB and the Fitmore® BS did not differ statistically (*p* = 0.919). Furthermore, when the stiffness of the stem was reduced, the strain load of the natural femur did not differ to the standard stem (CLS Spotorno®, *χ*^2^ = 5.21, *p* = 0.074) or the straight short stem (Ecofit Short®, *χ*^2^ = 0.04, *p* = 0.979).

### Strain analysis of the periprosthetic bone

Reducing the stiffness of the stem had the greatest effect on stress shielding in the calcar region.

Table 2 shows the results of the strain energy density (SED) change after virtual implantation for each stem-design/stiffness combination compared to a natural femur without prosthesis. Strain reduction was seen in medial regions of the bone after the implantation of full-bodied stems. In contrast, in the lateral regions, the SED increased for most of the other simulations. With a reduction of stem stiffness, in the medial bone regions, less stress shielding was induced. In contrast to the anatomical short stem, other stem types showed a lower strain gain in the lateral regions. Like the abovementioned findings, a clear reduction of the stress shielding effect appeared only in proximal medial bone regions.

**Table 2:** SED-changes [MPa] in Gruen’s zones (ROI) of all stem variations

SED-Changes [MPa]			
	FB	BS	SS
Fitmore®			
ROI 1	0.034	0.617	1.019
ROI 2	1.291	2.021	2.285
ROI 3	1.739	2.025	1.636
ROI 4	− 2.140	− 1.853	− 2.151
ROI 5	− 1.644	− 1.151	− 1.397
ROI 6	− 1.322	− 0.190	0.000
ROI 7	− 2.808	− 0.275	1.655
Ecofit® Short			
ROI 1	0.588	1.592	1.252
ROI 2	1.683	2.313	0.627
ROI 3	1.531	1.614	0.593
ROI 4	0.314	0.313	0.322
ROI 5	− 0.943	− 0.983	− 1.190
ROI 6	− 0.357	0.322	0.340
ROI 7	− 4.284	− 0.123	2.012
CLS Spotorno®			
ROI 1	0.173	0.989	1.015
ROI 2	0.197	0.293	− 0.294
ROI 3	− 0.443	− 0.769	− 0.938
ROI 4	− 0.567	− 0.942	− 1.025
ROI 5	− 3.319	− 2.697	− 2.884
ROI 6	− 2.782	− 1.548	− 1.793
ROI 7	− 3.257	− 0.858	0.397

**Fig. 3:**
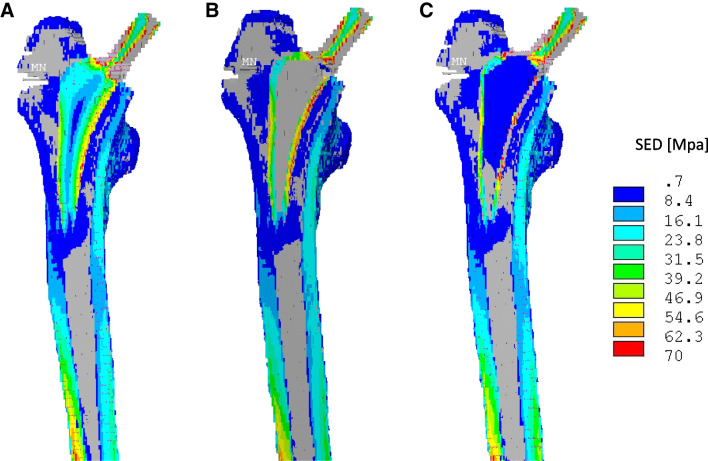
SED-Changes after virtual implantation of a Fitmore® stem [MPa]. The figure shows the distribution of strain energy in the femoral bone under force application. Reduction of the stress shielding shown by higher values is predominantly present in the medial regions. Force absorption by the stem decreases as the metal shell becomes thinner. **A** full-bodied original stem, **B** double-layer stem with a 1.32-mm big shell, and double-layer stem with a 0.66-mm small shell (**C**). Graphics were taken from Ansys®

Overall, these results showed that implantation of an anatomical short stem prosthesis together with a small titanium shell (Fitmore® SS) resulted in the most physiological loading effect (Fig. 3).

## Discussion

THA is a frequently performed orthopaedic procedure. Numerous approaches have been made to design the femoral stem in a way to mimic the physiological behaviour of a natural femur as close as possible. Therefore, with a multifactorial analysis, several combinations of stem length and stem stiffness were analysed with regard to their strain load and their consecutive effects on the femoral bone. The results of this study indicated that the combination of an anatomically designed short stem with a low stiffness might provide the most physiological strain transfer to the femoral side after THA.

However, the current study is limited by the virtual approach of THA stem implantation and the numerical method. It is unclear how data obtained from implantation simulations with FE software translate into the clinical setting without considering the biological effects of the bone. Our findings should be validated in DEXA or cadaver studies as a next step to obtain further data on the biomechanical physiology of the different stem types. This information might be a prerequisite for the design of a prospective clinical trial.

In this study, a previously validated set of FE data was used to analyse the impact of different stem designs and properties during THA on stress shielding and bone remodelling [2, 3, 11, 16, 17]. While dual X-ray absorptiometry (DEXA) can be useful for descriptive examinations with lower radiation doses than CT-based methods, DEXA cannot perform simulations like a virtual implantation [26].

Few mathematical approaches have been performed to transfer bone density values to elastic modulus [27–29]. Since FE models provide consistent bony strain patterns and are independent from the density-modulus relationship, it was possible to use a linear relationship according to studies from Ciarelli and colleagues [27]. Furthermore, common problems of a CT-based approach such as partial volume effects, fat errors, or metal artefacts had no impact on the results because the same dataset was used for each simulation [16]. An isolated resultant force on the hip centre verified by telemetric in vivo measurements was chosen because of widely varying effects of muscle forces on the SED [30].

Most research articles on THA employ a classification of zonal radiographic bone looseness described by Gruen and colleagues [24]. Since CT-based data sets provide high-resolution results, it became possible to analyse bones with a slice-by-slice technique including full three-dimensional information [26]. Like others, we used this approach to provide full slice resolution with a linear analysis [11, 17, 31].

Currently, there are multiple concepts to reduce stress shielding during THA [4, 5, 9, 11–15, 31]. In this context, stem design has become a research objective since the early days of THA. Many authors studied the behaviour of straight, tapered, and anatomical designs [12, 32–34]. Mostly, they found that anatomically designed stems produced the most physiological strain load for the bone remodelling processes [33]. However, with inconsistent study results, a real advantage for the clinical outcome remained questionable [32]. An established method to reduce stress shielding during THA by design variations has been found for the group of short stems [5, 35, 36]. Short stems with fixation techniques defined by osteotomy level are considered to provide a maximum of bone stock preservation until a first aseptic revision surgery is needed [7, 37]. However, the implantation of short stems has produced new problems including a potentially increased risk for periprosthetic fractures [6, 8]. In this context, it appears that consideration of stem design and length are not the one and only perfect solution for bone remodelling phenomenon after THA.

Therefore, other study groups analysed how stress shielding would be affected by using implants with a reduced stem stiffness such as low-stiffness composite prostheses like the Epoch® [13–15]. All those studies could describe reduced shielding when the rigidity of the femoral component was decreased [9, 11, 12, 19, 31, 32, 38]. However, new complications arose soon such as early failure of the stems [10]. Furthermore, interface shear stress between the implant and the periprosthetic bone increased with a lower stiffness and could lead to the loosening of the implant [11, 39]. Gross and Abel described that these effects could be reduced by the implantation of a hollow stem compared to a composite stem [11]. In this context, Bobyn and colleagues described that different components of stiffness must be considered for bone protection strategies in THA with stems [31]. They emphasised that the stiffness of the stem with an axial-, a compression-, a bending-, and a torsion-component must be considered when protection strategies are evaluated. Thereby, the component of the axial stiffness appears to be the most important parameter. If a stem was made hollow and more flexible, the axial stress was reduced most effectively if the so-called “threshold of flexibility” was reached with a very thin metal shell. A reduction in stem stiffness leads to a considerable effect on bone reactions, a phenomenon characterised as “threshold of flexibility”. However, the “threshold of flexibility” is only measurable when the elastic modulus of the implant falls below an individually defined threshold. In this context, other authors described the biomechanical behaviour of the femoral component during THA as a multifactorial function of fixation, material property, and design [40]. Bobyn and colleagues characterised the term of a “structural stiffness” which consists of stem geometry and elastic modulus. Both parameters were fitted into a close physiological window, the so-called “physioelasticity” [12].

Our findings appear to be the first effort to combine different current approaches to reduce stress-shielding effects in the proximal femur during THA in a FE analysis on in vivo data. Considering the multifactorial function, the best bone protection was found if an anatomic short stem with a reduced stiffness was used. Because only a very small titanium shell showed an effect on strain patterns, we could confirm the theory of a critical threshold from Bobyn and colleagues [31]. A combination of the different concepts for stem design, stem modulus, and stem length could further improve stem quality and lead to a most physiological stem.

## Conclusion

Based on the result of this study, a combination of a short and an anatomically designed stem with a low stiffness might provide a more physiological strain transfer during THA than other available stems without these properties. The biomechanical properties of the femoral component should be considered a multifactorial function including dimensions, design, and stiffness. Further research with translational approaches into clinical practice is needed to understand the impact of hip stem function on bone remodelling processes and to find the optimal stem with a minimal stress-shielding effect.

## Data Availability

Data is available by the corresponding author.
